# The TLR9 ligand CpG ODN 2006 is a poor adjuvant for the induction of de novo CD8^+^ T-cell responses in vitro

**DOI:** 10.1038/s41598-020-67704-0

**Published:** 2020-07-15

**Authors:** Laura Papagno, Nozomi Kuse, Anna Lissina, Emma Gostick, David A. Price, Victor Appay, Francesco Nicoli

**Affiliations:** 1 Centre d’Immunologie et des Maladies Infectieuses, Sorbonne Université, Institut National de la Santé et de la Recherche Médicale, 75013 Paris, France; 2 Center for AIDS Research, Kumamoto University, Kumamoto, 860-0811 Japan; 3 Division of Infection and Immunity, Cardiff University School of Medicine, Cardiff, CF14 4XN UK; 4 International Research Center of Medical Sciences, Kumamoto University, Kumamoto, 860-0811 Japan; 5 Department of Chemical and Pharmaceutical Sciences, University of Ferrara, 44121 Ferrara, Italy

**Keywords:** Adaptive immunity, Lymphocyte activation, Vaccines

## Abstract

Toll-like receptor 9 (TLR9) agonists have gained traction in recent years as potential adjuvants for the induction of adaptive immune responses. It has nonetheless remained unclear to what extent such ligands can facilitate the priming events that generate antigen-specific effector and/or memory CD8^+^ T-cell populations. We used an established in vitro model to prime naive precursors from human peripheral blood mononuclear cells in the presence of various adjuvants, including CpG ODN 2006, a synthetic oligonucleotide TLR9 ligand (TLR9L). Unexpectedly, we found that TLR9L induced a suboptimal inflammatory milieu and promoted the antigen-driven expansion and functional maturation of naive CD8^+^ T cells ineffectively compared with either ssRNA40 or 2′3′-cGAMP, which activate other pattern recognition receptors (PRRs). TLR9L also inhibited the priming efficacy of 2′3′-cGAMP. Collectively, these results suggest that TLR9L is unlikely to be a good candidate for the optimal induction of de novo CD8^+^ T-cell responses, in contrast to adjuvants that operate via discrete PRRs.

## Introduction

The development of effective cancer immunotherapies and vaccines will likely require interventions that generate potent CD8^+^ T-cell responses against tumor-associated antigens and/or neoantigens^1,2^. Adjuvants are critically important for such purposes, and in this context, recent efforts have focused on agonists that target Toll-like receptors (TLRs). Particularly robust adaptive immune responses have been observed in preclinical evaluations of costimulation via TLR9, which is typically activated by unmethylated CpG sequences present in microbial DNA^3,4^. Similarly, melanoma patients were found to harbor increased frequencies of human leukocyte antigen (HLA)-A*02:01-restricted Melan-A-specific CD8^+^ T cells after vaccination with the corresponding peptide adjuvanted by CpG ODN 2006, a synthetic oligonucleotide TLR9 ligand (abbreviated from hereon as TLR9L)^5,6^. However, these increments were founded on a background of established immunological memory, and as such, there is no a priori evidence to support the contention that primary CD8^+^ T-cell responses can be enhanced via TLR9. It is also important to note that inhibitory/tolerogenic effects have been attributed to TLR9Ls^7-11^.

In this study, we used an in vitro model to prime de novo CD8^+^ T-cell responses in the presence of various adjuvants, including CpG ODN 2006. Unexpectedly, we found that CpG ODN 2006 largely failed to enhance the expansion and functional maturation of naive antigen-specific CD8^+^ T cells, in contrast to a standard cocktail of inflammatory cytokines, ssRNA40 (TLR8L), or the stimulator of interferon genes (STING) ligand 2′3′-cGAMP.

## Results and discussion

**Figure 1:**
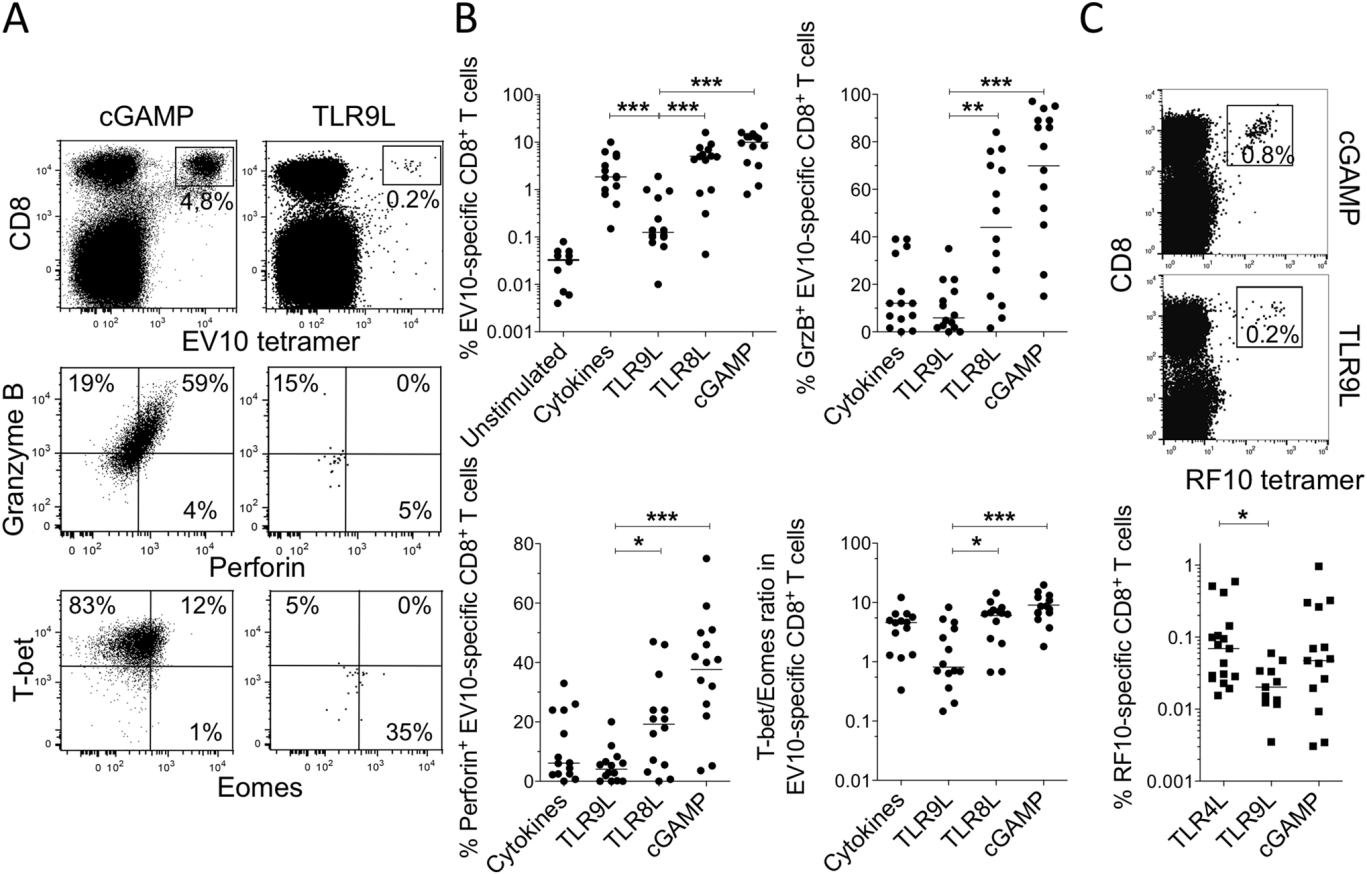
TLR9L does not enhance the expansion or functional maturation of naive antigen-specific CD8^+^ T cells. (**A**) Representative flow cytometry plots showing tetramer^+^ EV10-specific CD8^+^ T cells expanded in the presence of Flt3 ligand and either 2′3′-cGAMP or TLR9L (top) and intracellular expression of granzyme B and perforin (middle) or T-bet and Eomes (bottom) among the corresponding tetramer^+^ EV10-specific CD8^+^ T cells. Top: plots are gated on viable CD3^+^ events. Middle/bottom: plots are gated on tetramer^+^ EV10-specific CD8^+^ T cells. (**B**) Data summary across all priming conditions. Percentages and ratios were derived as shown in panel A. Each dot represents one HLA-A2^+^ donor per condition. Cytokines: TNF, IL-1β, IL-7, and PGE2. (**C**) Representative flow cytometry plots (top) and data summary (bottom) showing tetramer^+^ RF10-specific CD8^+^ T cells expanded as in panels A and B. Horizontal bars indicate median values. *p < 0.05, **p < 0.01, ***p < 0.001 (Mann–Whitney U test with Bonferroni correction for all conditions versus TLR9L).

To investigate the potential utility of CpG ODN 2006 (TLR9L) as a vaccine adjuvant, we used a previously optimized in vitro model that allows direct quantification of priming efficacy via the measurement of antigen-driven CD8^+^ T-cell expansion originating from naive precursors specific for Melan-A_26–35/A27L_ (EV10) restricted by HLA-A*02:01 (abbreviated from hereon as HLA-A2), which are readily accessible in standard preparations of peripheral blood mononuclear cells (PBMCs)^12,13^. Healthy donors were recruited for this study to minimize the likelihood of naturally occurring memory responses to EV10. We found that lower frequencies of EV10-specific CD8^+^ T cells were generated after 10 days in the presence of TLR9L compared with other adjuvants, namely a standard cocktail of inflammatory cytokines, ssRNA40 (TLR8L), or the STING ligand 2′3′-cGAMP (Fig. 1A,B). A similar pattern was observed on day 7, excluding the possibility of early induction and subsequent cell death, and on day 15, excluding the possibility of late induction (Supplementary Fig. S1A). Equivalent results were also obtained using a different neoantigen, HIV-1 Nef_138–147_ (RF10), restricted by a different allotype, HLA-A*24:02 (Fig. 1C). Moreover, EV10-specific CD8^+^ T cells primed in the presence of TLR9L expressed lower levels of the cytolytic molecules granzyme B and perforin compared with EV10-specific CD8^+^ T cells primed in the presence of TLR8L or 2′3′-cGAMP (Fig. 1A,B). Recent studies have shown that low expression levels of T-bet and high expression levels of Eomes are associated with functionally impaired CD8^+^ T cells^14,15^. In line with these findings, EV10-specific CD8^+^ T cells primed in the presence of TLR9L exhibited lower T-bet/Eomes ratios compared with EV10-specific CD8^+^ T cells primed in the presence of TLR8L or 2′3′-cGAMP (Fig. 1A,B and Supplementary Fig. S1B). All of these effects were abolished at lower concentrations of TLR9L (Supplementary Fig. S2).

**Figure 2:**
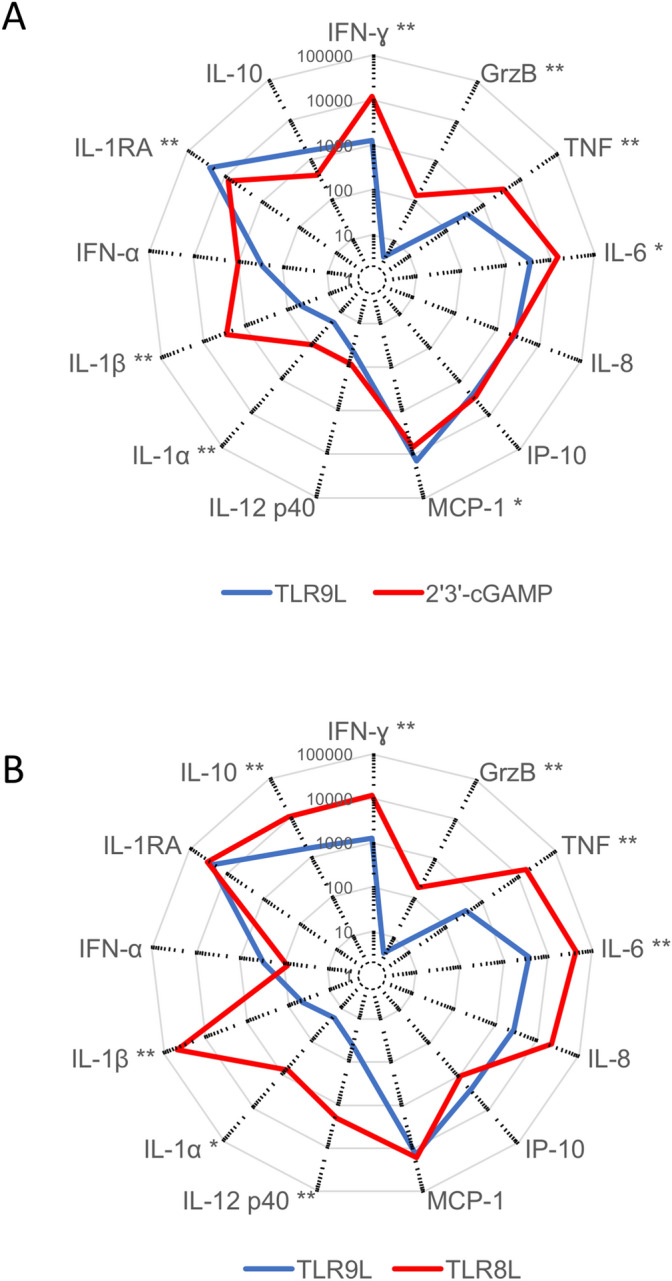
TLR9L induces a suboptimal inflammatory milieu among PBMCs. (**A**, **B**) Radar plots showing mean extracellular concentrations (pg/ml) of various chemokines and cytokines secreted by PBMCs (n = 10 donors) in response to overnight stimulation with TLR9L versus 2′3′-cGAMP (**A**) or TLR8L (**B**). * *p* < 0.05, ** *p* < 0.01 (Wilcoxon signed rank test with Bonferroni correction).

To assess the relationship between priming efficacy and the inflammatory milieu, we quantified various chemokines and cytokines secreted by PBMCs after overnight exposure to TLR9L, again using the comparators TLR8L and 2′3′-cGAMP (Fig. 2A,B). TLR9L induced the secretion of several inflammatory factors, including interleukin (IL)-8, interferon (IFN)-γ-induced protein (IP-10), and monocyte chemoattractant protein (MCP)-1, at levels equivalent to or greater than those induced by TLR8L or 2′3′-cGAMP. In contrast, key effector molecules, namely IFN-γ, tumor necrosis factor (TNF), and granzyme B, were secreted in much lower amounts after stimulation with TLR9L versus stimulation with either TLR8L or 2′3′-cGAMP. This pattern was replicated for cytokines with costimulatory effects known to play important roles in priming events, such as IL-1α, IL-1β, and, more selectively, IL-12. In addition, higher levels of IL-1RA were induced by TLR9L versus 2′3′-cGAMP. This soluble factor can dampen the activation of antigen-specific T cells^16^.

**Figure 3:**
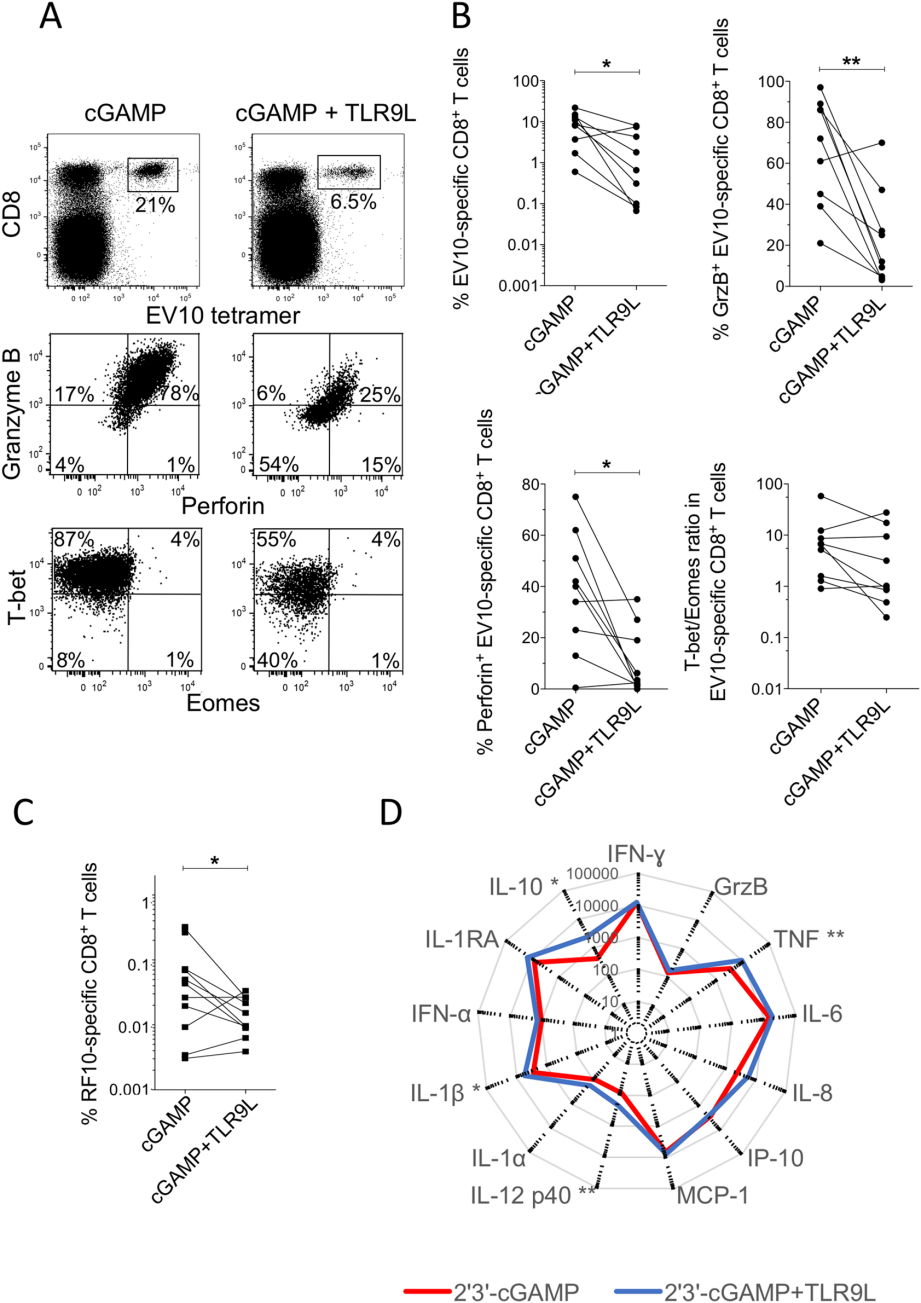
TLR9L inhibits 2′3′-cGAMP-mediated priming of naive antigen-specific CD8^+^ T cells. (**A**) Representative flow cytometry plots showing tetramer^+^ EV10-specific CD8^+^ T cells expanded in the presence of Flt3 ligand and 2′3′-cGAMP ± TLR9L (top) and intracellular expression of granzyme B and perforin (middle) or T-bet and Eomes (bottom) among the corresponding tetramer^+^ EV10-specific CD8^+^ T cells. Top: plots are gated on viable CD3^+^ events. Middle/bottom: plots are gated on tetramer^+^ EV10-specific CD8^+^ T cells. (**B**) Data summary across all priming conditions. Percentages were derived as shown in panel A. Each pair of dots represents one HLA-A2^+^ donor per condition. (**C**) RF10-specific CD8^+^ T cells were expanded in the presence of Flt3 ligand and 2′3′-cGAMP ± TLR9L. Details as in panel B. *p < 0.05, **p < 0.01 (Wilcoxon signed rank test). (**D**) Radar plots showing mean extracellular concentrations (pg/ml) of various chemokines and cytokines secreted by PBMCs (n = 10 donors) in response to overnight stimulation with 2′3′-cGAMP ± TLR9L. *p < 0.05, **p < 0.01 (Mann–Whitney U test).

To extend these findings, we tested the adjuvant effects of TLR9L in combination with 2′3′-cGAMP. These experiments were predicated on earlier work in mice, which showed that TLR9 and STING agonists acted synergistically to enhance various innate and adaptive immune responses^17^. In line with the induction of a suboptimal inflammatory milieu and previous studies in various experimental systems^7–11^, but counter to the notion of cooperative activity, we found that TLR9L suppressed the expansion and functional maturation of EV10-specific and RF10-specific CD8^+^ T cells adjuvanted by 2′3′-cGAMP (Fig. 3A–C). Although direct signaling via the STING pathway can inhibit cell proliferation, the priming efficacy of 2′3′-cGAMP relates primarily to the induction of type I IFNs via effects on DCs^13^. This latter process was likely impeded by TLR9L. We also found that TLR9L enhanced the production of IL-10 among otherwise subtle effects on 2′3′-cGAMP-induced patterns of chemokine/cytokine secretion in overnight assays with PBMCs (Fig. 3D). Of note, TLR9L has been shown to regulate plasmacytoid dendritic cell (pDC) responses to other immunostimulants via the induction of IL-10^18^, and high levels of IL-10 have been shown to inhibit the priming activity of 2′3′-cGAMP^13^. TLR9L can also upregulate the immunomodulatory enzyme indoleamine 2′3′-dioxygenase (IDO)^19,20^. However, our attempts to block these intermediaries using a purified anti-IL-10 monoclonal antibody or the IDO inhibitor D-1-methyl-tryptophan (D-1MT), respectively, failed to enhance the priming activity of TLR9L (Supplementary Fig. S3A).

To explore other potential mechanisms of suppression, we predepleted CD19^+^ cells, which include a population of DCs that acquire IDO-dependent regulatory functions in response to TLR9L^21^. In addition, we predepleted BDCA-3^+^ pDCs, which produce IFN-α in response to TLR9L^22^, and attempted to block the activity of IFN-α, which regulates many critical parameters involved in the genesis, maturation, and sustention of antigen-specific CD8^+^ T-cell populations^23^. None of these interventions enhanced the priming efficacy of TLR9L (data not shown and Supplementary Fig. S3B).

Collectively, these data show that TLR9L is a relatively poor adjuvant in vitro, at least compared with a standard cocktail of inflammatory cytokines, TLR8L, or 2′3′-cGAMP, all of which promoted the antigen-driven expansion and functional maturation of naive CD8^+^ T cells more effectively under otherwise identical conditions. TLR9L also inhibited the priming efficacy of 2′3′-cGAMP. This effect appeared to be independent of IDO, IFN-α, and IL-10. It should be noted that CpG ODN 2006 is a type B TLR9L. Further studies are therefore warranted to test the effects of type A and type C TLR9Ls, which are more potent inducers of IFN-α^3,4^. Moreover, our study was confined to the in vitro setting, which does not fully embrace the anatomical and physiological complexities of priming events in vivo, and further limited to a human system, which likely explains some of the discrepancies reported in mice^3^. Indirect effects may be especially pertinent in vivo. For example, TLR9L can stimulate B cells and CD4^+^ T cells to produce IgG2a and IFN-γ, respectively, which may promote the activation and expansion of CD8^+^ T cells^3,4^. In addition, we deliberately focused our investigations on the naive pool, excluding a formal evaluation of secondary responses, which may be preferentially amplified in the presence of TLR9L. Our results nonetheless suggest that CpG ODN 2006 is unlikely to be a good candidate for the optimal induction of de novo CD8^+^ T-cell responses, in contrast to TLR8L or 2′3′-cGAMP.

## Materials and methods

### Ethics

The use of human material was approved by the Comité de Protection des Personnes of the Pitié Salpétrière Hospital (France) and the Ethical Committee of Kumamoto University (Japan). Written informed consent was obtained from all donors in accordance with the Declaration of Helsinki.

### Peptides and tetramers

All peptides were synthesized at > 95% purity (Biosynthesis Inc.). The EV20 peptide (YTAAEELAGIGILTVILGVL, Melan-A_21–40/A27L_) was used for in vitro priming studies. Fluorochrome-labeled tetrameric complexes of HLA-A*02:01–EV10 (ELAGIGILTV; Melan-A_26–35/A27L_) and HLA-A*24:02–RF10 (RYPLTFGWCF, Nef_138–147_) were generated in-house as described previously^24,25^.

### In vitro priming of antigen-specific CD8^+^ T cells

PBMCs were isolated and cryopreserved from venous blood samples donated by healthy HLA-A2^+^ volunteers attending the Etablissement Français du Sang. Naive precursors specific for HLA-A2–EV10 were primed in vitro using an accelerated DC coculture protocol as described previously^12,26,27^. Briefly, thawed PBMCs were resuspended at 2.5 × 10^6^ cells/well in 48-well tissue culture plates containing AIM V medium (Thermo Fisher Scientific) supplemented with Flt3L (50 ng/ml; R&D Systems) to mobilize resident DCs. Predepletion of BDCA-3^+^ or CD19^+^ cells was achieved via magnetic selection using the corresponding MicroBeads (Miltenyi Biotec). After 24 h (day 1), the Melan-A peptide EV20 (1 µM) was added to the cultures, and DC maturation was induced under different adjuvant conditions, including: (1) a standard cocktail of inflammatory cytokines incorporating TNF (1,000 U/ml), IL-1β (10 ng/ml), IL-7 (0.5 ng/ml), and prostaglandin E2 (PGE2; 1 μM) from R&D Systems; (2) CpG ODN 2006 (TLR9L; 10 µg/ml) from InvivoGen; (3) ssRNA40 (TLR8L; 0.5 μg/ml) from InvivoGen; and (4) 2′3′-cGAMP (10 µg/ml) from InvivoGen. CpG ODN 2006 was combined in some experiments with purified anti-IL-10 (clone JES3-19F1; BioLegend) or D-1MT (Sigma-Aldrich). Negative control wells lacked EV20. On day 2, fetal bovine serum (FBS) was added at a final v/v ratio of 10%. Medium was replaced every 3 days thereafter with fresh RPMI 1640 containing 10% FBS. Unless stated otherwise, antigen-specific CD8^+^ T cells were characterized on day 10.

### Flow cytometry

The following directly conjugated monoclonal antibodies were used to stain CD8^+^ T cells: (1) anti-CD3–BV605 (clone SK7), anti-CD8–APC-Cy7 (clone SK1), anti-CCR7–PE-Cy7 (clone 3D12), and anti-granzyme-B–V450 (clone GB11) from BD Biosciences; (2) anti-CD27–Alexa Fluor 700 (clone O323) and anti-perforin–FITC (clone B-D48) from BioLegend; and (3) anti-CD45RA–PerCP-Cy5.5 (clone HI100), anti-T-bet–Alexa Fluor 647 (clone 4B10), and anti-Eomes–PE-eFluor 610 (clone WD1928) from eBioscience. Dead cells were eliminated from the analysis using LIVE/DEAD Fixable Aqua (Thermo Fisher Scientific). Expression of T-bet and Eomes was measured using a Transcription Factor Buffer Set (BD Biosciences). Intracellular staining for granzyme B and perforin was compatible with this procedure^28^. Data were acquired using an LSR Fortessa (BD Biosciences) and analyzed with FlowJo software version 9.3.7 (Tree Star Inc.). The gating strategy is shown in Supplementary Fig. S4.

### Measurement of soluble factors

Chemokines and cytokines were measured using a Luminex T200 instrument in combination with a human Bio-Plex Immunoassay Kit (Bio-Rad). All concentrations were determined as the mean of two replicates after background subtraction.

### Statistics

Univariate statistical analyses were performed using nonparametric tests in Prism software version 8.4.2 (GraphPad). Significance was assigned at *p* < 0.05.

## Supplementary information

Supplementary file1 (DOCX 766 kb)

## Data Availability

All primary data generated during the course of this study are available from the corresponding authors upon reasonable request.
