# Supplementary material for: The TLR9 ligand CpG ODN 2006 is a poor adjuvant for the induction of de novo CD8+ T-cell responses in vitro
Source: Sci Rep. 2020 Jul 15;10:11620. doi: 10.1038/s41598-020-67704-0 (PMC7363897; doi:10.1038/s41598-020-67704-0)
Supplement: Supplementary file 1 — Supplementary file1 (DOCX 766 kb) [file 41598_2020_67704_MOESM1_ESM.docx]

**SUPPLEMENTARY INFORMATION**

**The TLR9 ligand CpG ODN 2006 is a poor adjuvant for the induction of *de novo* CD8^+^ T-cell responses *in vitro***

Running title: Limited adjuvant efficacy of TLR9L

Laura Papagno^1^, Nozomi Kuse^2^, Anna Lissina^1^, Emma Gostick^3^, David A. Price^3^, Victor Appay^1,4,*^ and Francesco Nicoli^1,5,*^

^1^Sorbonne Université, Institut National de la Santé et de la Recherche Médicale, Centre d’Immunologie et des Maladies Infectieuses, 75013 Paris, France

^2^Center for AIDS Research, Kumamoto University, Kumamoto 860-0811, Japan

^3^Division of Infection and Immunity, Cardiff University School of Medicine, Cardiff CF14 4XN, Wales, UK

^4^International Research Center of Medical Sciences, Kumamoto University, Kumamoto 860-0811, Japan

^5^Department of Chemical and Pharmaceutical Sciences, University of Ferrara, Ferrara 44121, Italy

* These authors contributed equally to this work.

**Supplementary Figure S1.** Kinetics of naive antigen-specific CD8^+^ T-cell expansion in the presence of TLR9L. EV10-specific CD8^+^ T cells were expanded in the presence of Flt3 ligand and either a standard cocktail of inflammatory cytokines or TLR9L. (**A**) Percentages of tetramer^+^ EV10-specific CD8^+^ T cells. (**B**) T-bet/Eomes ratios among tetramer^+^ EV10-specific CD8^+^ T cells. Each dot represents one HLA-A2^+^ donor per condition. Cytokines: TNF, IL-1β, IL-7, and PGE2.

**Supplementary Figure S2.** Expansion and functional maturation of naive antigen-specific CD8^+^ T cells in the presence of different concentrations of TLR9L. EV10-specific CD8^+^ T cells were expanded in the presence of Flt3 ligand and either a standard cocktail of inflammatory cytokines or TLR9L at the indicated concentrations. Graphs show percentages of tetramer^+^ EV10-specific CD8^+^ T cells and intracellular expression of granzyme B or perforin among the corresponding tetramer^+^ EV10-specific CD8^+^ T cells. Each dot represents one HLA-A2^+^ donor per condition. Horizontal bars indicate median values. Cytokines: TNF, IL-1β, IL-7, and PGE2.

**Supplementary Figure S3.** The adjuvant efficacy of TLR9L cannot be enhanced by blocking IL-10, inhibiting IDO, or predepleting CD19^+^ cells from PBMCs**.** (**A**) EV10-specific CD8^+^ T cells were expanded in the presence of Flt3 ligand and either a standard cocktail of inflammatory cytokines or TLR9L ± anti-IL-10 (50 μg/ml) or D-1MT (4 μM). Graphs show percentages of tetramer^+^ EV10-specific CD8^+^ T cells and intracellular expression of granzyme B, perforin, or T-bet/Eomes among the corresponding tetramer^+^ EV10-specific CD8^+^ T cells. (**B**) EV10-specific CD8^+^ T cells were expanded from either unmanipulated or CD19^+^ cell-depleted (CD19d) PBMCs in the presence of Flt3 ligand and either a standard cocktail of inflammatory cytokines or TLR9L. Data are presented as in panel A. Each dot represents one HLA-A2^+^ donor per condition. Horizontal bars indicate median values. Cytokines: TNF, IL-1β, IL-7, and PGE2.

**Supplementary Figure S4.** Flow cytometric gating strategy for the identification of EV10-specific CD8^+^ T cells. Bottom: representative flow cytometry plots showing intracellular expression of granzyme B and perforin or T-bet and Eomes among tetramer^−^ versus tetramer^+^ EV10-specific CD8^+^ T cells.
